# Photothermal Phase Change Energy Storage Materials: A Groundbreaking New Energy Solution

**DOI:** 10.34133/research.0460

**Published:** 2024-08-20

**Authors:** Linghang Wang, Huitao Yu, Wei Feng

**Affiliations:** School of Materials Science and Engineering, Tianjin University, Tianjin 300350, P. R. China.

## Abstract

To meet the demands of the global energy transition, photothermal phase change energy storage materials have emerged as an innovative solution. These materials, utilizing various photothermal conversion carriers, can passively store energy and respond to changes in light exposure, thereby enhancing the efficiency of energy systems. Photothermal phase change energy storage materials show immense potential in the fields of solar energy and thermal management, particularly in addressing the intermittency issues of solar power. Their multifunctionality and efficiency offer broad application prospects in new energy technologies, construction, aviation, personal thermal management, and electronics.

## Introduction

The global energy transition requires new technologies for efficiently managing and storing renewable energy. In the early 20th century, Stanford Olshansky discovered the phase change storage properties of paraffin, advancing phase change materials (PCMs) technology [1]. Photothermal phase change energy storage materials (PTCPCESMs), as a special type of PCM, can store energy and respond to changes in illumination, enhancing the efficiency of energy systems and demonstrating marked potential in solar energy and thermal management systems. In 2016, 178 parties signed the Paris Agreement, committing to limit global temperature rise to below 2 °C. This agreement greatly accelerated the development of renewable green energy technologies. Since 2017, research on PTCPCESMs has significantly increased. In 2023, China included PTCPCESMs in policy support, recognizing their key role in improving the efficiency, durability, and sustainability of new energy technologies [2].

## Solar Energy Challenges and PCM Solutions

Solar energy is abundant, but because of the intermittent nature of sunlight, solar thermal technology faces substantial issues with narrow application time frames and unstable energy utilization. Traditional solar systems cannot operate outside of sunlight hours, often resulting in low utilization rates, as seen with solar collectors and solar dryers [3]. PTCPCESMs can alter their physical state or properties by utilizing solar radiation, absorbing excess heat during peak sunlight periods, and releasing heat when solar intensity is lower or at night, thereby achieving energy storage and controlled release. Consequently, PTCPCESM technology is considered one of the most effective solutions to address the intermittency problem of solar energy.

## Main Characteristics of PTCPCESMs

PCMs can absorb or release a substantial amount of heat near their melting points through phase changes, storing or releasing energy. These characteristics make them suitable for use as thermal storage media in solar collection systems or as working substances in heat pump systems, providing various functionalities in multiple ways [4]. In thermodynamics, energy conversion during phase changes involves changes in system entropy and thermal radiation losses. The latent heat absorbed or released by PCMs during melting or solidification is directly related to changes in the system’s disorder. However, during this process, some energy is lost as thermal radiation, depending on the material’s surface characteristics and environmental conditions. In addition, PCMs also have drawbacks such as low thermal conductivity, low photothermal conversion efficiency, and leakage during the phase change process.

PTCPCESMs consist of PCMs and various carriers—organic, inorganic, carbon-based composite, and metal-based—that often encapsulate the PCMs in microcapsules or porous materials. These carriers are primarily focused on enhancing photothermal conversion rates, while also improving thermal conductivity, sealability, and the control of thermal radiation intensity in PCMs. For commonly used PTCPCESM, the photothermal conversion efficiency is required to be above 50% to 70%. The thermal conductivity typically ranges from 0.2 to 0.5 W/m·K, and for composite materials with enhanced thermal conductivity, it can reach 1 to 2 W/m·K. The phase change enthalpy of PTCPCESM usually ranges from 150 to 250 J/g. In this context, porous materials are often used as carriers for infusing PCM. Photothermal conversion is generally achieved through 3 mechanisms: molecular vibrational heating, localized plasmonic heating, and nonradiative relaxation heat release [5].

In carbon-based materials and some organic polymers, the ease of electron excitation from π to π* orbitals, followed by heat release through vibronic electron coupling when these electrons return to their ground state, facilitates molecular vibrational heating. For instance, Atinafu et al. [6] developed a graphene derived from solid sodium acetate to enhance the photothermal conversion efficiency, thermal conductivity, and energy storage capacity of PCMs. The reduction in supercooling increased the composite material’s energy storage capacity by 157.6 kJ/kg, which is 101.4% higher than expected. Graphene, with its high thermal conductivity and photothermal responsiveness, effectively controls thermal radiation and absorbs solar light from visible to near-infrared. Its 2-dimensional structure enhances thermal transfer and surface area, promoting rapid heat distribution between PCMs and carriers.

Metal-based materials, such as gold nanoparticles and MXene, enhance light–matter interactions and break the traditional diffraction limit through localized surface plasmon resonance by confining incident light to nanoscale dimensions. The excitation of plasmons greatly enhances the electromagnetic fields near the structure, greatly increasing absorption and scattering at the resonance frequency. These properties allow metal nanostructures to have enhanced light collection and focusing capabilities. Fan et al. [7] reported on a novel polyethylene glycol/Ti_3_C_2_T*_x_* layered phase change composite material, which exhibits strong absorption in the ultraviolet-visible-near-infrared region due to the localized surface plasmon resonance effect of Ti_3_C_2_T*_x_* nanosheets, achieving up to 94.5% photothermal conversion efficiency under solar irradiation.

When semiconductor materials or certain special organic molecules are excited by photons, they generate electron–hole pairs, which can release energy either radiatively or nonradiatively. In nonradiative relaxation, the excited electrons transfer their energy to the lattice, generating phonons and causing a rise in local semiconductor temperatures. Ge et al. [8] studied a light-driven microfluidic control device that utilizes light-responsive alkoxylated grafted azobenzene PCM to collect, transmit, and utilize energy in low-temperature environments. This device effectively controls temperature through photothermally driven heat release under conditions as low as −40 °C and achieves a high energy density of 380.76 J/g even at −63.92 °C. The thermal effect is primarily due to light-induced molecular isomerization, a nonradiative relaxation process. When light excites azobenzene, the molecules shift from one conformation to another, allowing the light-responsive switch material to control its structure and thus regulate thermal radiation intensity.

Appropriate carrier selection significantly enhances the thermal conductivity of PCMs. Wei et al. [9] demonstrated this using cellulose aerogel and molybdenum disulfide as carriers, which increased the thermal conductivity of PCMs by 138%. Through improvements in PCM performance by different carrier materials, PTCPCESMs demonstrate substantial potential in enhancing energy efficiency and meeting diverse application needs.

## Potential Applications of PTCPCESMs in New Energy Technologies

Besides solar systems, PTCPCESMs find extensive applications in the construction industry. Typically, PTCPCESMs are integrated into walls, roofs, and floors to maintain stable indoor temperatures without external energy input, thereby reducing energy consumption [10]. With technological advancements, we believe that PTCPCESMs will be widely applied in various emerging fields such as new energy vehicles, personal thermal management, aerospace, and electronic information.

**Fig. 1.**
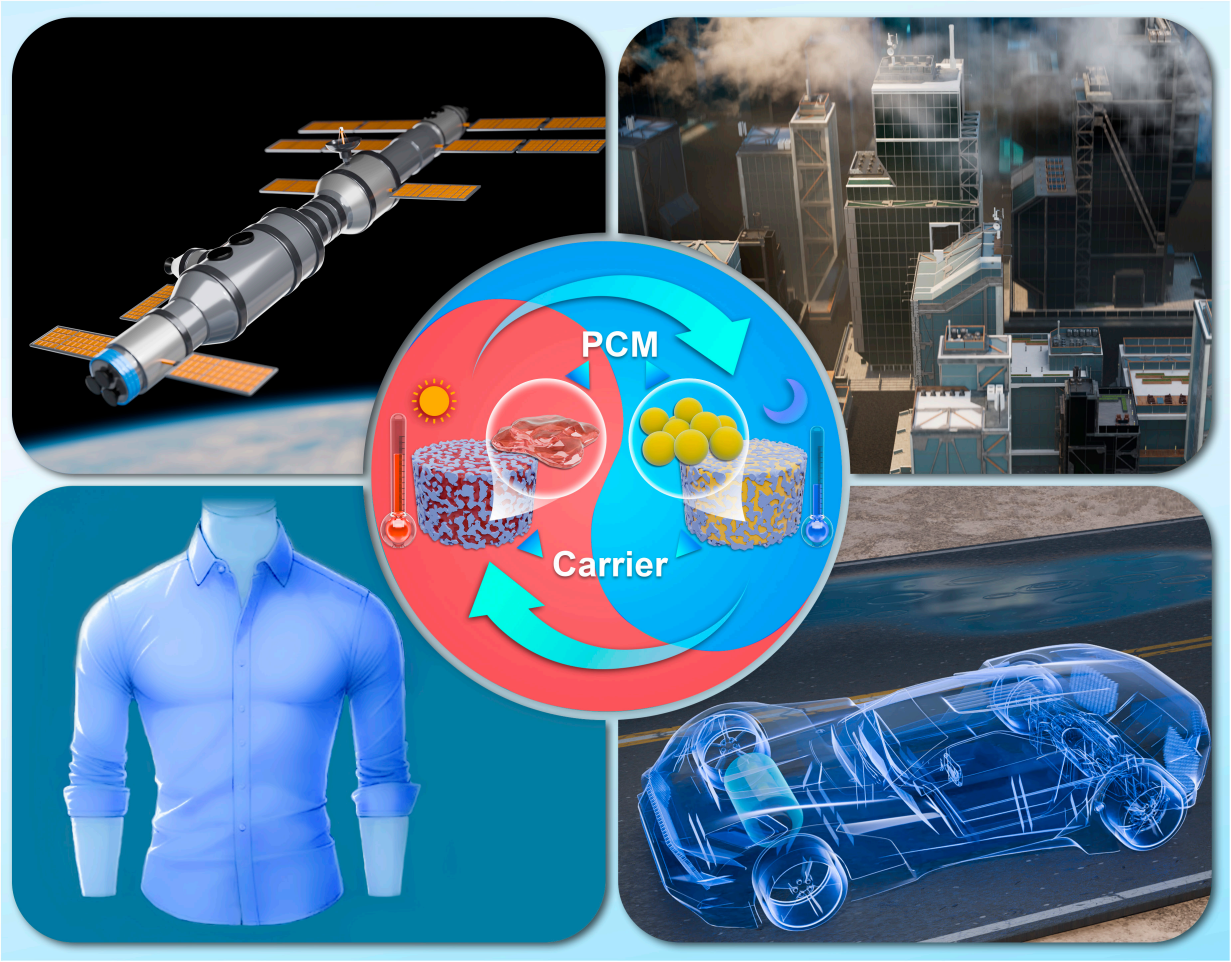
Prospects for the application of PTCPCESMs.

As illustrated in Fig. 1,when PCMs are combined with carriers, they utilize the photothermal conversion properties of the carriers to achieve energy storage. During periods of abundant sunlight, the carriers convert solar energy into heat, inducing a phase change in the PCMs and storing energy. In the absence of sunlight, the PCMs release the stored heat, providing a thermal buffering effect. In electric vehicles, PTCPCESMs can balance and manage cabin heat between day and night, improving comfort without requiring additional energy. They can also be incorporated into adjustable clothing to automatically regulate the wearer’s body temperature by absorbing and releasing heat, enhancing comfort and energy efficiency. In deep space exploration, PTCPCESMs can maintain spacecraft components and instruments within operational temperature ranges, protecting sensitive instruments and reducing the energy needed for heating and cooling systems. Furthermore, PTCPCESMs can absorb and store heat generated by high-power electronic devices during high activity and release it during low temperatures, ensuring a stable internal environment. The multifunctionality and efficiency of PTCPCESMs suggest their increasingly important role in modern energy and material technologies, providing sustainable and efficient energy solutions across various industries.

## Conclusion

PTCPCESMs have considerably improved thermal conductivity and photothermal conversion efficiency and have partially addressed leakage issues. However, they still face challenges like low mechanical strength, poor interfacial compatibility, and slow environmental response. Future innovations should focus on (a) developing PTCPCESMs with higher photothermal conversion efficiency, (b) exploring PTCPCESMs with precisely adjustable thermal radiation intensity of PCMs, (c) improving the encapsulation system of PTCPCESMs to enhance their durability and leakage prevention capabilities, (d) introducing support frameworks or rigid sealing materials to enhance the mechanical strength of the carriers and composite materials, (e) improving the interfacial compatibility and thermal conductivity between PCMs and carriers through chemical modification; and (f) continuously researching high-thermal-conductivity PTCPCESM systems to optimize performance across diverse applications.

PTCPCESMs are transforming energy management across multiple fields. Their ability to effectively store and manage thermal energy makes them indispensable in the ongoing transition to sustainable energy practices. As the world continues to make technological breakthroughs in solar energy, electric vehicles, green buildings, and space exploration, PCMs will play a crucial role in achieving a sustainable and efficient future.
